# Dimension-Dependent Phenomenological Model of Excitonic Electric Dipole in InGaAs Quantum Dots

**DOI:** 10.3390/nano12040719

**Published:** 2022-02-21

**Authors:** Petr Steindl, Petr Klenovský

**Affiliations:** 1 Department of Condensed Matter Physics, Faculty of Science, Masaryk University, Kotlářská 267/2, 61137 Brno, Czech Republic; steindl@physics.leidenuniv.nl; 2 Huygens-Kamerlingh Onnes Laboratory, Leiden University, P.O. Box 9504, 2300 RA Leiden, The Netherlands; 3 Czech Metrology Institute, Okružní 31, 63800 Brno, Czech Republic

**Keywords:** electric dipole, quantum dots, InGaAs, **k**·**p** method, electronic structure

## Abstract

Permanent electric dipole is a key property for effective control of semiconductor quantum-dot-based sources of quantum light. For theoretical prediction of that, complex geometry-dependent quantum simulations are necessary. Here, we use k·p simulations of exciton transition in InGaAs quantum dots to derive a simple geometry-dependent analytical model of dipole. Our model, discussed here, enables reasonably good estimation of the electric dipole, caused in quantum dot by the elastic strain, including an externally induced one. Due to its apparent simplicity, not necessitating elaborate and time-consuming simulations, it might after experimental verification serve as a preferred choice for experimentalists enabling them to make quick estimates of built-in and induced electric dipole in quantum dots.

## 1. Introduction

Due to their discrete energy levels with the molecular-like character [1] and strong quantum confinement of electrons and holes in all dimensions [2], semiconductor quantum dots (QDs) serve as an excellent solid-state platform for a number of appealing applications. Among others, they may be used as gain material for semiconductor lasers [3,4] or as building blocks of nonvolatile universal memory, so-called QD-Flash [5,6]. Due to near unity quantum efficiency and external-field tuneability, QD optical transitions are often used as sources of quantum light [7,8] in advanced quantum communication and computation schemes [9,10]. This application demands well-defined transitions energies and control of QDs’ interaction with the charge environment.

Permanent electric dipole (*p*) is one of the key properties of semiconductor quantum dots connecting their electronic structure with optical activity. Since it directly relates to the separation of electron and hole wavefunction [11,12], it can be used for identification of the type of QD spatial confinement. More importantly, the dipole plays significant role in fine-tuning of QDs’ emission energies through in-plane applied electriec field via quantum Stark effect [13,14]. Such control of emission energies is used in cavity quantum electrodynamics for tuning QD emission into resonance with optical cavity [8,15], or for control of photon indistinguishability from remote quantum emitters [16,17].

Even though theoretical predictions of the electric dipole of QDs with realistic shape currently exist, they are typically based on complex single-particle quantum simulations requiring a definition of the full heterostructure, strain energy minimalization in that, and quantification of the related strain and piezoelectricity-induced changes of the confinement potentials [2]. In principle, such calculation can be done with atomistic precision within empirical pseudopotential [18,19] or tight-binding models [2,20], but since that approach is computational-heavy, it is typically used for QDs with a rather small volume. To account for effects at macroscopic dimensions, tight-binding models are typically replaced with computationally lighter approximate semiempirical methods based on macroscopic properties of the heterostructure, such as k·p simulations [21,22]. Because performing such advanced simulations requires years of experience with complex software tools [23], hands-on approximations of geometry-dependent electric dipole for QDs are practical but nonexistent to date. Therefore, in this study, we extend model from our recent work [24] on QD-geometry-induced changes of electric dipole and discuss a phenomenological model of the electric dipole motivated by analytical estimation of *p* of 1D quantum well [25,26] and found by systematic analysis of a set of k·p simulations.

## 2. Modeling of Electric Dipole

In our previous work [24], we showed that the electrical polarization P and the corresponding built-in dipole moment in stress-tuned ${\mathrm{In}}_{x}{\mathrm{Ga}}_{1-x}$As/GaAs QDs [27] are mainly influenced by one of the second-order terms in the expansion of P into strain (η), the dominant term is denoted by the coefficient $B_{124}$. Based on that observation, an approximate formula to reproduce *p* as a function of applied shear in-plane stress $\sigma_{xy}^{\mathrm{appl}}$ was derived

(1) $$p/e\propto A^{\mathrm{QD}}\left(\sigma_{xy}^{\mathrm{appl}}+\sigma_{xy}^{\mathrm{pre}}+\sigma_{xy}^{\mathrm{QD}}\right),$$

where $\sigma_{xy}^{\mathrm{pre}}$ represents off-diagonal component of the (symmetric) in-plane prestress induced in the heterostructure by bonding on the piezoelectric actuators for external stress tuning, $\sigma_{xy}^{\mathrm{QD}}$ is the in-plane component of hydrostatic stress in QD, and *e* denotes the elementary charge. That relation allows splitting changes of *p* into contributions driven by the stress-tuning and terms purely related to build-in QD dipole effected only by QD lattice relaxation-induced hydrostatic stress. Since the scaling factor $A^{\mathrm{QD}}$ can be written as [24]

(2) $$A^{\mathrm{QD}}=C^{\mathrm{QD}}\frac{B_{124}\;\eta_{H}^{\mathrm{QD}}}{eG},$$

we can further separate QD geometry, represented by geometry-dependent scaling factor $C^{\mathrm{QD}}$, from hydrostatic strain effects. In Equation (2), $B_{124}$ represents the second-order term in the expansion of P; *G* and $\eta_{H}^{\mathrm{QD}}$ are the shear modulus and the hydrostatic strain in QD, respectively. Values of parameters $B_{124}$ and *G* for a specific QD’s material composition *x* are estimated by linear interpolation of parameters listed in Table 1.

Since the parameter $C^{\mathrm{QD}}$ reflects the quantum confinement effect on the quasiparticle position in QD, quantum simulations are needed for its quantification. In this work, we extract the parameter $C^{\mathrm{QD}}$ from simulations of *p* calculated for a truncated cone shape ${\mathrm{In}}_{x}{\mathrm{Ga}}_{1-x}$As QD by eight-band k·p approximation. In our simulations, we keep In-Ga alloy distribution constant at value showing good agreement in emission energy, fine-structures splitting of QD exciton, and *p* between theory and experiment (x=0.45) [24] and vary only QD spatial dimensions, i.e., QD’s top (*t*) and bottom (*b*) diameter, and height (*h*).

The full calculation flow of simulations discussed in this work was as follows. First, the geometry of the QD structure was defined on a rectangular grid including the spatially dependent material constituents. Thereafter, the strain field in and around QD was found by minimizing the strain energy, followed by calculation of the effect of resulting strain on the confinement potential was then calculated using the Bir-Pikus Hamiltonian with positionally dependent material parameters listed in the supplementary materials of [24]. In the next step, the self-consistent solution of single-particle Schrödinger and Poisson equations including the effect of piezoelectric fields up to second order in η were calculated by Nextnano3 simulation suite [23]. The obtained single-particle states within the envelope function method based on an eight-band k·p approximation are then used for accounting for multiparticle interactions, including direct and exchange Coulomb interaction [31,32,33]. Restricting ourselves only to bright exciton transition, we use the multiparticle corrected electronic states for *p* calculation, i.e., the distance between electron- and hole-wavefunctions center of mass. Assuming that the geometrical dependency of $C^{\mathrm{QD}}$ for any QD shape will for laterally large dots converge to the dependency $p\propto h^{4}$ derived for infinitely large 1D potential well of thickness *h* [25,26], we can quantify the dipole correction for the lateral quantum confinement by further analysis of $c^{\mathrm{QD}}$ as

(3) $$C^{\mathrm{QD}}=c^{\mathrm{QD}}h^{4}.$$

**Figure 1:**
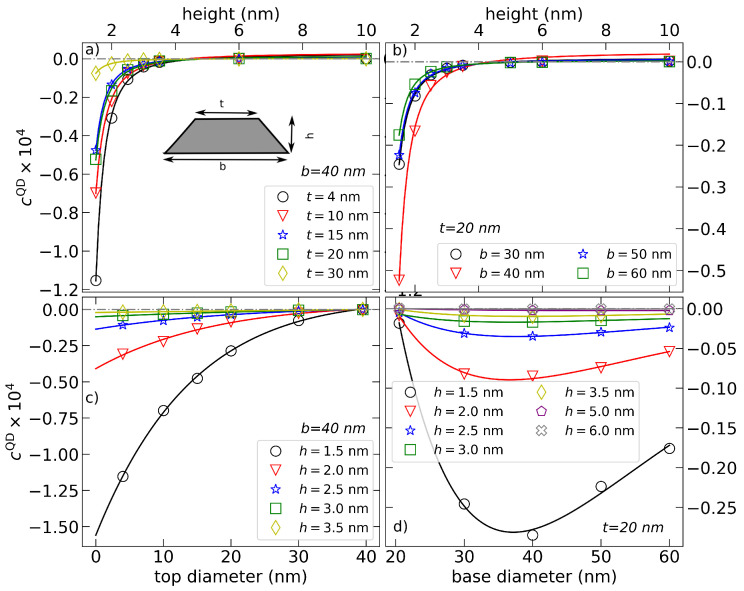
Comparison of $c^{\mathrm{QD}}$ (multiplied by factor $10^{4}$) extracted from k·p simulations (symbols) with the model Equation (4) (curves) for QD with fixed b=40 nm and varying *t* and *h* [panels (**a**) and (**c**)], and QD with fixed t=20 nm and varying *b* and *h* [in (**b**,**d**)], respectively.

By careful cross-analysis of simulated sets of *p* for varying *t*, *b*, and *h* presented in the panels of Figure 1, we find that $c^{\mathrm{QD}}$ can be approximated by

(4) $$c^{\mathrm{QD}}=A\left(b-t\right)\exp\left(B\;\frac{\left|b-t\right|}{h}\right)+D.$$

**Figure 2:**
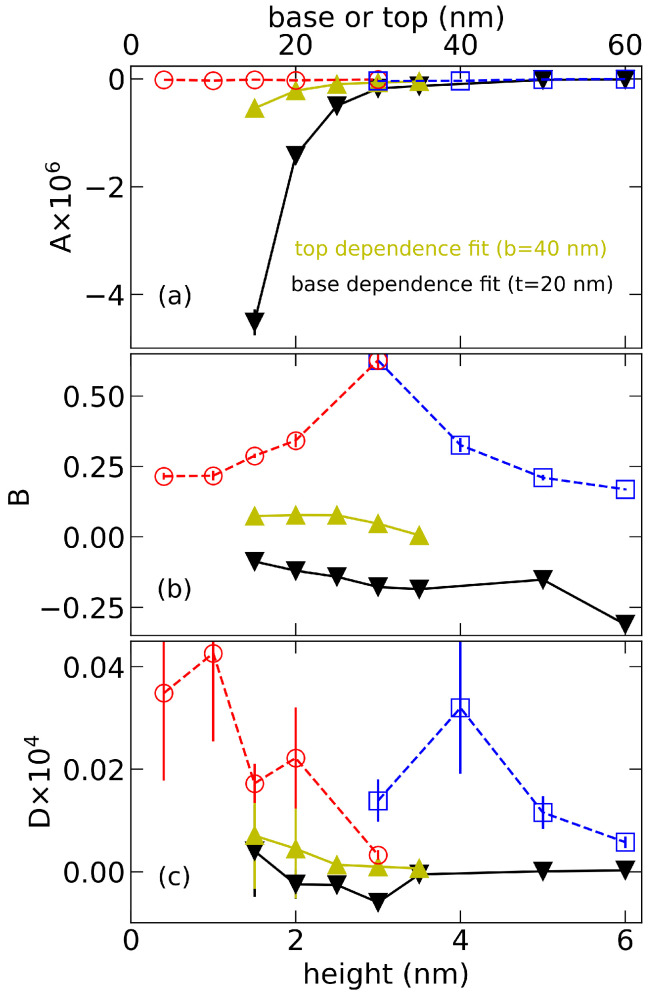
Fitting parameters A [panel (**a**)], B [panel (**b**)], and D [panel (**c**)] from analysis of $c^{\mathrm{QD}}$ given in Figure 1 by Equation (4). Parameters *A* and *D* are multiplied by factors $10^{6}$ and $10^{4}$, respectively, for the sake of better visibility. Parameters extracted from fits of the data dependency of $c^{\mathrm{QD}}$ on base (top) are plotted here as a function of height with black (yellow) triangles. Similarly, parameters extracted from height dependency are plotted as a function of base (top) diameter in blue (red).

Now, we analyze the fitted parameters *A*, *B*, and *D*, shown in Figure 2. Parameter *A* in Figure 2a) is found to be independent of the QD lateral dimensions, while it clearly retains a residue dependency on QD height. This dependency is stronger for smaller QDs and exponentially disappears for taller dots. Contrary, parameter *B* in Figure 2b) shows strong dependency only on lateral dimensions, where it increases proportionally to the dot top diameter B∝−1/t and decreases with its base diameter as B∝1/b. Finally, $c^{\mathrm{QD}}$ for laterally big QDs converges to the parameter *D*, represented in Figure 2c). Even though we initially assumed *D* to be geometry-independent, it still shows a dependency for small QDs. That dependency, together with high statistical error from our fits, could be related to an artefact of rough simulation grid in k·p calculations, where the electron wavefunction with the tendency to be located at the top of the dot would need a finer grid for better convergence.

The presented model merges together into one simple analytical expression two methods used for estimation of QD electric dipole. On the one hand, it is an extension of the already existing, explicitly geometry-dependent model of *p* derived for 1D well [25,26], used widely also in QD research, on lateral confinement. On the other hand, our model is developed from complex k·p simulations of realistic QDs, where the electron and hole eigenstates of QD, and thus also *p*, are corrected for piezoelectricity, stress, and many-body effects. Note that due to the form of Equation (4), our model also correctly describes the sign of *p* in QD with reverted vertical orientation.

The model relies only on the validation of the approximation from Equation (1), derived under assumptions: (i) the QD has type-I confinement, (ii) it consists from piezoelectric material with dominant second-order term $B_{124}$, and (iii) the hydrostatic strain around QD is mostly driven by the material mismatch between the dot and the substrate $\eta_{H}^{\mathrm{QD}}$. Because the model is proportional not only to QD geometry but also to material parameters, it can be, therefore, expected to be applicable also for other III-V QD systems [14,34,35,36,37] with type-I confinement where the electric dipole is aligned only along the dot growth direction.

## 3. Conclusions

In conclusion, we show a phenomenological analytical model describing the excitonic electric dipole of ${\mathrm{In}}_{0.45}{\mathrm{Ga}}_{0.55}$As QDs with geometry-dependent parameters quantifying quantum confinement effect. The presented simple model of electric dipole agrees with dipole simulation done with complex eight-band k·p approximation, which recently fully described single-quantum dot experiments [24]. The presented model, being a 3D confinement extension of model derived for 1D well [25,26], needs to be further tested on a combination of morphology and single QD spectroscopy data set. Thereafter, due to its strong dependency on individual spatial properties of studied QDs, it can serve as a rough but straightforward estimate of QD’s spatial dimensions directly from spectroscopic measurements without necessity of additional information taken from, e.g., STM measurements [38].

## Figures and Tables

**Table 1:** Parameter values used in k·p calculation and in Equation (2).

	$B_{124}$ ($C/m^{2}$) [28]	*G* (GPa)
InAs	−4.1	19.00 [29]
GaAs	−3.8	32.85 [30]

## Data Availability

The data that support the findings of this study are available upon reasonable request from the authors.
